# Role of Nucleotide P2 Receptors in the Immune System: Focus on Effector T Cells

**DOI:** 10.3390/cells14181467

**Published:** 2025-09-19

**Authors:** Romuald Brice Babou Kammoe, Chakib Hamoudi, Fawzi Aoudjit

**Affiliations:** 1 Infectious and Immune Diseases Axis, CHU de Quebec Research Center, Laval University, Quebec City, QC G1V 4G2, Canada; romuald.babou@crchudequebec.ulaval.ca (R.B.B.K.); chakib.hamoudi@crchudequebec.ulaval.ca (C.H.); 2 ARThritis Research Center, Laval University, Quebec City, QC G1V 4G2, Canada; 3 Department of Microbiology-Infectiology and Immunology, Faculty of Medicine, Laval University, Quebec City, QC G1V 0A6, Canada

**Keywords:** P2 receptors, effector T cells, human Th17 cells, autoimmunity

## Abstract

The emergence of purinergic signaling has given ATP and other extracellular nucleotides a whole new perspective. This concept refers to the fact that extracellular nucleotides released by most cells act as signaling molecules via specific membrane receptors called purinergic receptors, thus regulating several cellular functions. Although purinergic signaling has been well studied in several physiological systems, recent work has shown that this signaling pathway is also essential in the immune system. In this review, we will discuss the complex role of purinergic signaling in the regulation of effector T cell functions, including migration, differentiation, and activation. We will also cover the role of P2 receptors in the development of autoimmune diseases. Understanding how P2 receptors regulate effector T cells will likely further our understanding of protective immunity and immune pathogenesis and may lead to new therapeutic approaches and agents in immune diseases

## 1. Introduction

Nucleotides are widely dispersed molecules that are essential for regulating various biological processes. They are the basic building blocks of nucleic acids and play crucial functions such as cofactors, energy intermediates, and allosteric modulators. Furthermore, they also act as ubiquitous triggers for the release of intracellular messengers [1,2,3]. Burnstock introduced the term “purinergic signalling” in the 1970s to emphasize the role of the nucleoside adenosine and the purine nucleotides ATP and ADP in the intricate process of intercellular communication [4]. Initially, there was considerable skepticism due to the unusual possibility that cells, which are thought to be in a state of normal physiological functioning, might release ATP into the extracellular environment [1,2]. Purinergic signaling has undergone significant expansion since then, and it is now clear that extracellular nucleotides, particularly ATP, are widely dispersed signaling molecules released during neurotransmission and in response to various cellular stressors, including osmotic swelling, mechanical deformation, hypoxia, and cytotoxic substances [1,2]. Following their initial classification as neurotransmitters, ATP and its dephosphorylated derivatives have been found to serve as signaling molecules in various physiological processes [2].

In a multicellular system, purinergic signaling enables individual cells to adjust their reactions, ultimately benefiting the organism as a whole [5]. Three fundamental components make up purinergic signaling: (i) mechanisms that generate and release ATP into the pericellular space; (ii) purinergic receptors that detect released ATP and its metabolites and trigger intracellular signals that control cell functions; (iii) mechanisms that stop purinergic signaling through cellular re-uptake, enzymatic breakdown of ATP, or simple diffusion of ATP and its metabolites away from cells [5].

The release of nucleotides occurs under both physiological and pathological conditions, either through cell lysis or via other mechanisms, including the exocytosis of ATP-containing vesicles and nucleotide-permeable membrane channels, such as connexin and pannexin semichannels [1,6,7]. Once in an extracellular environment, ATP acts as a signaling molecule via so-called purinergic type 2 (P2) receptors to regulate cellular functions. Purinergic signaling can act in an autocrine manner, i.e., on the cells that released it, or in a paracrine manner on neighboring cells. P2 receptors comprise two subfamilies: the P2Y and P2X receptors [8,9]. P2Y receptors are G-protein-coupled metabotropic receptors that, in addition to ATP, can bind other nucleotides such as ADP or UTP. There are eight functional P2Y receptors, namely P2Y_1_, P2Y_2_, P2Y_4_, P2Y_6_, P2Y_11_, P2Y_12_, P2Y_13_, and P2Y_14_ [10,11]. P2X receptors, on the other hand, are ionotropic receptors that bind exclusively to ATP and act as ion channels, inducing ionic movements, including Na^+^, K^+^, and Ca^2+^ ions [11]. The P2X receptor subfamily comprises seven subtypes (P2X1–P2X7) with varying sensitivities to ATP [12,13].

**Figure 1:**
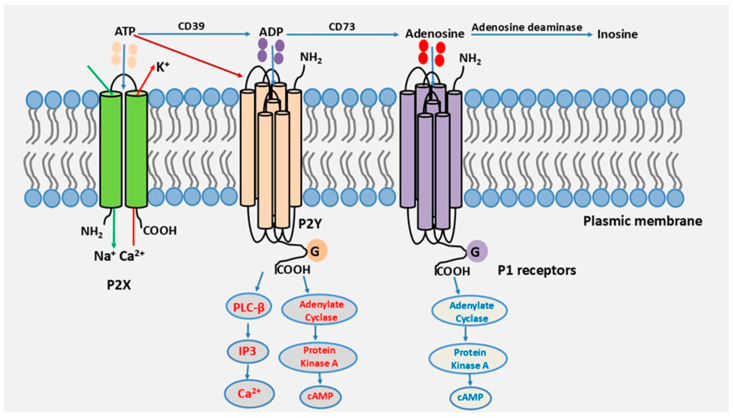
The purinergic signaling cascade.

After exerting its effect on P2 receptors, ATP comes into contact with ectonucleotidases expressed on the surface of most cell types, with distinct distribution profiles [14]. ATP will be sequentially degraded by CD39 and CD73 nucleotidases, leading to the subsequent generation of adenosine, a biologically active metabolite that activates P1 receptors [15,16,17]. P1 receptors belong to the family of G protein-coupled transmembrane receptors. There are four subtypes of adenosine receptors, each with distinct affinity and signaling properties: A_1_, A_2A_, A_2B_, and A_3_ [18,19]. These receptors participate in terminating the action of ATP and P2 receptors, often exhibiting anti-inflammatory effects. Of note, adenosine can also be degraded by adenosine deaminase to inosine. A schematic representation of the purinergic signaling cascade is shown in Figure 1.

## 2. P2 Receptor Expression in the Immune System

P2 receptors are expressed, either constitutively or following cellular activation, on a range of cells, including immune cells [20]. Several studies have demonstrated the importance of extracellular ATP in the activation processes and regulation of various immune system cell functions [20,21]. Furthermore, ATP released by damaged cells acts as a danger signal. Indeed, when cellular damage occurs, for example, following tissue injury or when cells become apoptotic or necrotic, the ATP released into the extracellular space induces several cellular responses, including the recruitment of innate immune cells to the injured site, to ensure clearance of the danger [22,23,24]. Released ATP can have autocrine and paracrine effects not only on innate immune cells, such as neutrophils, macrophages, dendritic cells, and natural killer (NK) cells, but also on adaptive immune cells, including T cells [14,21]. This review will focus on the role of purinergic signaling in T cells, with a particular emphasis on effector T cells.

## 3. Role of P2 Receptors in T Cell Activation and Chemotaxis

Purinergic signaling has been much less studied in T cells compared to other leukocytes. However, T cells can also release ATP, which facilitates autocrine T cell activation and intercellular communication [25,26,27]. Several stimuli induce ATP release from T cells, including during T cell activation upon antigen recognition of naive T cells by the T cell receptor (TCR), mechanical stimulation, membrane deformation, and osmotic stress, among others. ATP released by semichannels, particularly pannexin-1, plays a critical role in the activation of naive T cells [28]. Several studies have demonstrated that T cells can express multiple P2 receptors, including P2X1, P2X4, P2X5, P2X7, and P2Y_11_ [17,20,25,27,29,30,31,32,33,34]. Notably, the P2X5 receptor is non-functional in humans, although it appears to be functional in some African Americans [35].

Initial studies in the context of T cell activation showed that TCR activation induces ATP release and autocrine stimulation of P2X1, P2X4, and P2X7 receptors. Activation of these receptors contributes to increased intracellular calcium, the activation of the transcription factor NFAT, and IL-2 expression, all of which are associated with CD4^+^ T cell proliferation [22,23,25,36]. From a functional point of view, activation of the TCR/CD28 complex of CD4^+^ T cells during immunological synapse formation induces ATP production from mitochondria. It causes the translocation of pannexin-1 semichannels, as well as P2X1, P2X4, and P2X7 receptors to the immunological synapse to orchestrate autocrine purinergic signaling, leading to T cell activation, cytokine production and proliferation [33].

However, more recent studies have shown that CD4^+^ T cell activation and proliferation are primarily and strongly dependent on the P2X4 receptor, but not on the P2X1 and P2X7 receptors [25,34]. Rather, the P2X1 receptor has been implicated in maintaining the metabolism of quiescent T cells, which prepares them for antigen recognition, but is not required for T cell activation [37]. P2X7 receptors are found at the immunological synapse and are also uniformly distributed on the cell surface during lymphocyte activation. This distribution would allow T cells to remain sensitive to ATP generated by tissues and cells that are not directly involved in antigen presentation [14]. P2X7 receptor plays a role in the apoptosis of naive CD4^+^ T cells through a mechanism involving ERK1/2 signaling [38,39]. In addition to P2X receptors, the P2Y_11_ receptor supports T lymphocyte activation by directing mitochondrial trafficking to the immunological synapse [40]. On the other hand, studies have shown that the P2Y_11_ receptor has a protective effect against the apoptotic effects of the P2X7 receptor in naive CD4^+^ T cells, via a mechanism that involves inhibiting the P2X7 receptor’s ability to form nonspecific pores [41,42]. In addition to the P2Y_11_ receptor, although the P2Y_1_ receptor is weakly expressed in T cells, a study has shown that it can also participate in the activation of naive T cells [27].

Taken together, these studies suggest that different P2 receptors play distinct roles in T cell physiology, depending on the steps involved in T cell activation, with a significant role assigned to the P2X4 receptor [43,44]. These studies also suggest the existence of two distinct autocrine purinergic signaling systems in T cells: one system that facilitates T cell vigilance and involves the P2X1 receptor, and a second system that regulates T cell functional responses following antigen recognition and is predominantly dependent on the P2X4 receptor [37,43].

T cells must migrate to encounter antigen-presenting cells (APCs) and to execute their varied functions in immune defense and inflammation [25]. In addition to T cell activation, purinergic signaling is also essential for chemotaxis and migration of T cells towards APCs in response to the chemokine CXCL12 or SDF-1α [25]. To this end, P2X4 and P2Y_11_ receptors have been identified as critical. These receptors act synergistically to orchestrate the metabolic program that regulates T cell polarization and migration [25,27,45]. Indeed, P2X4 receptor will localize in front of the cell in the direction of migration by amplifying the chemokine signal through autocrine stimulation. At the same time, the P2Y_11_ receptor participates in the retraction of the uropods of migrating T cells [45].

Most of the above studies have been conducted with peripheral blood CD4^+^ T cells and in the context of naïve CD4^+^ T cell activation. Since the primary focus of this review is on effector T cells and autoimmunity, we will not elaborate further on the associated mechanisms. These have been elegantly addressed by the research group of W.G. Junger, and the readers are invited to check these papers for more details [14,26,27,37,40].

## 4. Role of P2 Receptors in Effector T Cells

While the role of P2 receptors in T cell activation is established, few studies, especially human studies, have investigated how purinergic receptors regulate effector T cell functions. Several studies have addressed this issue by utilizing animal models of autoimmunity. Th17 cells represent a crucial effector T cell subset in the development of autoimmune diseases [46,47], and growing evidence suggests that P2 receptors are crucial for the functions of Th17 cells and in the regulation of autoimmune diseases. In the following, we will discuss these findings as well as address other effector T cell subsets, including Th1, Th2, and FoxP3-positive T regulatory cells (Tregs).

### 4.1. Role of P2 Receptors in Th17 Cells Differentiation and Activation

Naïve CD4^+^ T cells are polarized towards the Th17 lineage during antigen recognition and in the presence of specific cytokines including IL-6, TGFβ, IL-23; and IL-1β, which lead to the expression of the transcription factor RORc, the master regulator of the Th17 lineage, which in turn, lead to the production of IL-17 cytokine [48,49]. Under certain circumstances like in response to IL-6, Tregs can also differentiate into inflammatory Th17 cells [50,51].

Little work has been conducted on how P2 receptors regulate Th17 cells. Nevertheless, early work reported that P2 receptors could influence Th17 cells differentiation indirectly by acting on dendritic cells during antigen presentation. ATP, via the P2X7 receptor, acts through dendritic cells to promote Th17 cells generation in a mouse model of ovalbumin-induced asthma [52]. Similarly, P2X7 receptor was shown to enhance Th17 cell differentiation and collagen-induced arthritis in mice by enhancing the production of Th17 cell-polarizing cytokines by dendritic cells [22]. In a mouse model of experimental autoimmune encephalomyelitis (EAE), it was found that P2Y_6_ receptor signaling inhibits the production by dendritic cells of the Th1 and Th17 cells polarizing cytokines IL-12 and IL-23, respectively, and leads to the inhibition of the differentiation of Th1 and Th17 cells subpopulations and to the reduction in EAE severity [53]. Furthermore, during the acute inflammation of inflammatory bowel disease, IL-6 increases ATP production and activates the P2X7 receptor on Tregs, which induces their conversion into Th17 cells [54]. Along these lines, in an allergy mouse model, mast cells can counteract Tregs partially through IL-6-mediated Tregs differentiation towards Th17 cells [55].

Although the above studies performed in mouse models of inflammation provided evidence of the role of P2 receptors in the regulation of Th17 cells, it is not clear if P2 receptors can directly regulate effector T cell differentiation and whether this can occur in human effector T cells. In this context, we recently reported that the P2X4 receptor is a key player in the differentiation of human Th17 cells. We have shown that human Th17 cells exhibit a P2 receptor expression profile similar to that of naive CD4^+^ T cells, although their expression was higher on Th17 cells. Thus, Th17 cells express mostly P2X4, P2X5, P2X7, and P2Y_11_ receptors along with weak levels of P2X1, and we have identified the P2X4 receptor as a critical pathway for the differentiation and activation of human Th17 cells [34]. Indeed, inhibition of these receptors showed that only P2X4 receptor inhibition interfered with the differentiation and generation of human Th17 cells from naive CD4^+^ T cells isolated from peripheral blood. In contrast, P2X4 receptor inhibition did not affect the differentiation of human Th1 cells, a T cell subpopulation that also plays a vital role in the development of autoimmune diseases [56]. We also examined whether the different receptors regulated cellular expansion and proliferation during the differentiation process. 75% fewer differentiated Th17 cells were obtained when the P2X4 receptor was blocked in comparison with control cells in which the receptor was not blocked. However, the P2X7 and P2Y_11_ receptor inhibitors did not affect the cellular expansion of Th17 cells but decreased Th1 cell growth. These findings suggest that P2X4 receptor is necessary for the proliferation of both human Th1 and Th17 cells but only affects Th17 cells’ differentiation. The requirement of the P2X4 receptor for both Th1 and Th17 cells proliferation is thought to be due to the fact that naive CD4^+^ T cells require P2X4 receptor activation for proliferation [25].

**Figure 2:**
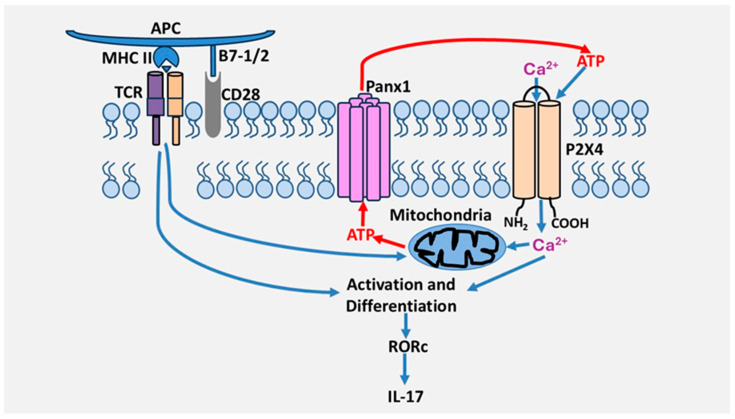
Proposed model of the role of the P2X4 receptor in the regulation of Th17 cells. T cell activation through the TCR/CD28 complex in the presence of Th17-polarizing cytokines induces ATP release via pannexin-1 channels, which leads to P2X4 receptor activation, which is necessary for TCR/CD28-induced RORc expression levels, Th17 cell differentiation, and IL-17 production.

We also investigated the involvement of P2 receptors in the reactivation of Th17 cells, which refers to the capacity of polarized/differentiated Th17 cells to produce IL-17. To this end, receptor antagonists were not added during differentiation but only during the reactivation of polarized Th17 cells with anti-CD3/CD28 antibodies. Following this activation, we found that inhibition of the P2X4 receptor with 5-BDBD decreased IL-17 production but had no effect on IFN-γ production. On the other hand, inhibition of P2X7 and P2Y_11_ receptors did not affect the output of either cytokine. Furthermore, combined inhibition of P2X4, P2X7, and P2Y_11_ receptors did not produce a more potent inhibition on IL-17 production than inhibition of the P2X4 receptor alone. These results suggest that the P2X4 receptor is the most critical P2 receptor in IL-17 production. This is supported by the fact that the P2X4 receptor is involved in the expression of retinoic acid-receptor-related orphan receptor c (RORc) but not T-bet. These two transcription factors are the primary regulators of IL-17 and INF-γ gene expression, respectively [34]. These studies suggest that purinergic signaling differentially regulates helper T cell programs and identify for the first time the P2X4 receptor as a primary receptor in the activation and differentiation of human Th17 lymphocytes. However, it remains to be determined by which mechanisms the P2X4 receptor contributes to the differentiation of human Th17 cells. Interestingly, the P2X4 receptor also regulates human Th2 activation, suggesting it could also be important in Th2-dependent immunity [34]. A model by which the P2X4 receptor promotes Th17 cell differentiation and activation is depicted in Figure 2.

### 4.2. Role of P2 Receptors in Th17 Cell Migration

After their differentiation in lymphoid organs, Th17 cells migrate through extracellular matrix of target tissues to reach inflammatory sites where their reactivation leads, to the production of IL-17, which orchestrates the inflammatory response and tissue damage [57,58]. Although the role of P2 receptors has been demonstrated in the chemotaxis of immune cells including T cells [25], their involvement in Th17 cells’ adhesion and migration through extracellular matrix has not been studied. T cell migration through extracellular matrix requires the intervention of integrins, a large family of α/β transmembrane receptors that play a key role in cell–cell interactions and cell adhesion [59,60].

**Figure 3:**
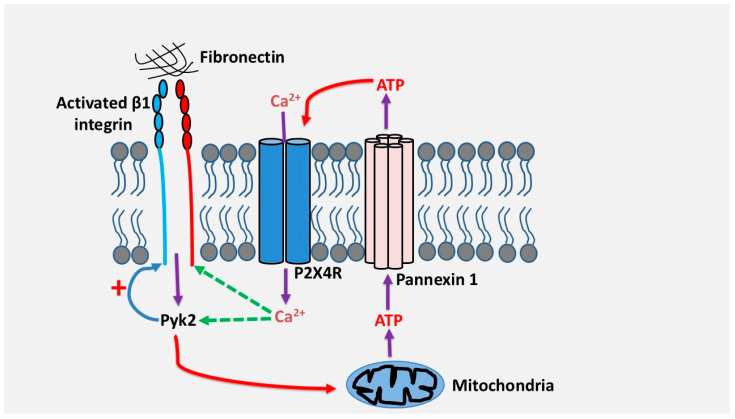
Proposed model by which the P2X4 receptor promotes adhesion and migration of human Th17 lymphocytes via β1 integrins. Cell adhesion to fibronectin via β1 integrin activates PYK2, leading to ATP release through pannexin-1. ATP/P2X4 receptor signaling triggers calcium influx, which in turn activates PYK2, resulting in enhanced β1 integrin activation, further promoting adhesion and inducing migration of Th17 cells.

We have demonstrated in our laboratory that the adhesion and migration of human Th17 cells through fibronectin, a major extracellular matrix protein whose expression is strongly increased in inflammatory tissues, depends on the signaling of the purinergic receptor P2X4 [61]. Adhesion to fibronectin via β1 integrins, α4β1 and α5β1, induces a sustained release of ATP in quantities sufficient to activate the P2X4 receptor [61]. This occurred via mitochondrial ATP and pannexin-1 channels. Inhibition of the P2X4 receptor but not of the P2X7 and P2Y_11_ receptors inhibited the adhesion and migration of Th17 cells through fibronectin, suggesting the existence of an autocrine activation pathway between β1 integrins and the P2X4 receptor [61]. Mechanistically, the P2X4 receptor, but not other receptors, strengthens the activation of β1 integrins, which is important to enhance cell adhesion and migration [61]. This likely occurred through increased intracellular calcium via P2X4 receptor and activation of the focal adhesion kinase PYK2, which can be activated by calcium, and which plays a critical role in cell adhesion and migration. On the other hand, we found that PYK2, was also essential for fibronectin-induced ATP release suggesting a positive feedback loop of activation regulation between integrin β1, P2X4 receptor and PYK2 in human Th17 cells [61]. Based on these results, the model below (Figure 3) illustrates how P2X4 receptor regulates Th17 cell migration in fibronectin. It remains to be determined if Th17 cells and other leukocytes also depend on purinergic signaling and P2X4 receptor to migrate in different contexts and invade other matrices like collagens or laminins. However, human Th17 cells migrate through laminin by using P2X4 receptor (unpublished observations) suggesting that the implication of purinergic receptors in leukocyte migration and invasion could be a general mechanism. This is reinforced by studies that reported that activation of P2Y_1_ and P2Y_12_ receptors induced calcium-dependent activation of platelet integrin αIIbβ3 in megakaryocytes [62] and that Thy-1-induced astrocyte migration depend on activation of the P2X7 receptor by integrin αvβ3 [63].

Despite the above studies, it remains unclear which P2 receptors regulate Th17 cell migration in mice. A previous study demonstrated that the P2X4 receptor promotes T cell recruitment to the lungs in a mouse model of orthotopic lung transplantation [25].

## 5. P2 Receptors in Effector T Cells and Autoimmune Diseases

Autoimmune diseases are characterized by loss of immune tolerance, the presence of autoreactive lymphocytes, and the generation of autoantibodies against self-components and persistent inflammation leading to tissue damage [64].

It is now widely accepted that P2 receptors play a crucial role in regulating the inflammatory response by activating immune cells [17,20,65]. The propagation cascade of the inflammatory response and tissue damage can lead to the release of a significant amount of ATP and the stimulation of P2 receptors on immune cells, resulting in a positive feedback mechanism that extends the inflammation response [66]. Furthermore, given the role of purinergic signaling in effector T cells strongly suggest that P2 receptors are important players in the development of autoimmune diseases.

### 5.1. Rheumatoid Arthritis

The role of purinergic signaling in the development of rheumatoid arthritis was suggested in the 1990s, following the proposal of adenosine as a therapeutic option [67]. Most studies have shown that ATP in rheumatoid arthritis acts via the P2X7 receptor. Indeed, the expression and function of the P2X7 receptor are increased on immune cells isolated from patients with arthritis [68,69,70].

Additionally, the P2X7 receptor enhances the production of cathepsins by macrophages, which may promote bone resorption associated with rheumatoid arthritis [71]. Finally, the promoting effect of P2X7 receptor on Th17 cell differentiation was investigated in CD4^+^ T cells co-cultured with dendritic cells in the collagen-induced arthritis mouse model [22]. The results of this study revealed that blocking the P2X7 receptor with antagonists decreases the development of arthritis in mice by inhibiting Th17 cells differentiation, which occurs after blocking the expression of Th17 cell polarizing cytokines (IL-1β, TGF-β1, IL-23, and IL-6) by dendritic cells [22]. However, one study showed that monocytes and lymphocytes from arthritic patients exhibit reduced expression of the P2RX7 gene [69]. Furthermore, we found that the P2X7 receptor is not required for human Th17 cell differentiation [34]. In addition, clinical trials targeting the P2X7 receptor have had no effect in the treatment of rheumatoid arthritis, suggesting that the P2X7 receptor is insufficient for the development of rheumatoid arthritis and that other receptors are also important [72]. Indeed, several studies have shown that the P2X7 receptor requires the presence of the P2X4 receptor for optimal functioning [44,73,74,75,76].

The P2X4 receptor has been linked to joint pain [77] and the upregulation of human Th17 cell differentiation and migration [34,61]. Along these lines, we also showed that the blockade of P2X4 receptor decreased the production of IL-17 by 30–40% in activated effector/memory CD4^+^ T cells isolated from the peripheral blood of patients with rheumatoid arthritis [34]. In contrast, inhibition of the P2X7 receptor had no effect on IL-17 production, which may explain why P2X7 receptor inhibitors failed in clinical trials. The blockade of P2X4 and P2X7 receptors also did not affect the production of IFN-γ by rheumatoid effector/memory CD4^+^ T cells, demonstrating no influence on Th1 function [34]. These results suggest that the P2X4 receptor is a pathogenic pathway in rheumatoid arthritis, primarily through the upregulation of Th17 cell function [34]. Moreover, the P2X4 receptor antagonist, 5-BDBD, also reduced the severity of collagen-induced arthritis in mice by inhibiting Th17 activation [34]. Along these lines, the P2X4 receptor antisense oligonucleotides reduced synovial inflammation in the collagen-induced mouse model of arthritis by modulating the serum levels of IL-1β, TNF-α, IL-6, and IL-17 [78]. Together, these results combining mice and human studies suggest that the P2X4 receptor is a critical pathway in the development of rheumatoid arthritis, arguing in favor of assessing P2X4 receptor inhibitors in clinical trials for the treatment of rheumatoid arthritis.

Regarding P2Y receptors, the P2Y_11_ receptor has been implicated in the cytokine-induced inflammatory response of patients’ primary fibroblast-like synoviocytes [79]. Indeed, blockade of this receptor with the specific antagonist NF340 inhibited IL-1β-induced expression of TNF-α and IL-6 [79]. The P2Y_6_ and P2Y_12_ receptors participate in bone resorption during osteoporosis via osteoclast activation [80,81], while the P2Y_14_ receptor has the opposite effect by stimulating osteogenesis via osteoblasts [82,83]. However, the role of these P2Y receptors in animal models of arthritis has not been investigated.

### 5.2. Systemic Lupus Erythematosus

The P2X7 receptor is involved in systemic lupus erythematosus through the activation of the NLRP3 inflammasome and increased production of IL-1β and IL-18 by lupus patient-derived macrophages [84,85], thereby contributing to the cardiovascular, cutaneous, and renal manifestations of lupus [86,87,88]. Th1 and Th17 cells exhibit higher expression of the P2X7 receptor in lupus patients compared to controls, and P2X7 receptor levels on Th17 cells correlate with diseases activities of both lupus and rheumatoid arthritis, suggesting an important role for P2X7 in those two diseases [89]. However, in experimental murine lupus, the P2X7 receptor plays a protective role by limiting the expansion of pathological T follicular helper cells through the induction of pyroptosis and decreasing the generation of autoreactive antibodies [90]. These contrasting studies could be due to the presence of different P2X7 receptor variants in humans, each with distinct functional characteristics [91].

As B cells and plasma cells are critical effectors in lupus, it has recently been shown that bone marrow plasma cells (PCs) sense extracellular ATP via P2X4 receptor [92]. In this study, the authors demonstrated that bone marrow PCs utilize the ligand-gated purinergic ion channel P2X4 receptor to sense extracellular ATP released by bone marrow osteoblasts through the gap junction protein Pannexin 3 (Panx3) [92]. Mutating the P2X4 receptor in developing B-lineage cells using Mb1-Cre resulted in lower serum antibody titers and significantly reduced numbers of bone marrow PCs, demonstrating that the P2X4 receptor is necessary to establish normal bone marrow PC populations [92]. The P2X4 receptor specific inhibitor 5-BDBD abrogated the impact of extracellular ATP on PCs in vitro and depleted bone marrow PCs in vivo [92]. P2X4 receptor blockade also reduced autoantibody titers and kidney disease in two mouse models of systemic lupus erythematosus characterized by serum antibodies against double-stranded DNA (dsDNA) and progressive proteinuria [92]. While the role of P2X4 receptor on Th17 cells in lupus has not been examined, it is likely from the studies on rheumatoid arthritis that inhibition of Th17 cells also contribute along with the inhibition of plasma cells to the protective effect of P2X4 receptor antagonist in lupus.

### 5.3. Multiple Sclerosis

Genetic mutations in the P2X4 and P2X7 receptors, which result in a functional loss of these receptors, have been associated with familial multiple sclerosis [93]. Along these lines, activating P2X4 receptor promoted the remyelination response and improved clinical signs of experimental EAE in mice, whereas inhibition or genetic deletion of the P2X4 receptor exacerbates the disease [94].

Conflicting results have been reported regarding the function of P2Y_12_ in the development of EAE. One study reported that P2Y_12_ receptor-deficient mice were protected from EAE [95]. Indeed, deficiency of the P2Y_12_ receptor led to a sharp decrease in the percentage of Th17 cells, accompanied by decreased IL-17A production and a low mRNA level of Th17 cell-related genes [95]. In contrast, a second study found that the absence of P2Y_12_ exacerbated the disease [96]. In this study, the deletion of the P2Y_12_ receptor boosted the expression of IL-17A in the serum and the proportion of Th17 cells in the spleen and central nervous system. Loss of P2Y_12_ significantly increased the production of the Th17 cells polarizing cytokine IL-23 in contrast to the wild-type (WT) BMDCs [96]. Flow cytometry analysis also indicated that the culture supernatant from P2Y_12_ receptor-deficient DCs promoted the differentiation of more naïve CD4^+^ T cells into Th17 cells [96]. Furthermore, a pro-inflammatory environment, which damages neurons, has been associated with loss of P2Y_12_ receptor expression in microglia from multiple sclerosis patients [97].

Loss of P2Y_6_ receptor expression has also been associated with exacerbating EAE in mice [53]. Increased expression of IL-12 and IL-23 was detected in P2Y_6_ receptor-deficient bone marrow-derived dendritic cells compared to controls [53]. This increased expression was correlated with enhanced Th1/Th17 cells polarization by mature dendritic cells [53]. These data demonstrate that P2Y_6_ receptor functions as a crucial regulator of DC maturation, and its deletion results in worsened EAE [53].

Together, these studies tend to suggest that P2 receptors may play a protective role in the development of multiple sclerosis.

### 5.4. Inflammatory Bowel Diseases (IBD)

P2X7 receptor plays a vital role in the development of inflammatory bowel diseases. It upregulates the production of pro-inflammatory cytokines (Myd88, NF-κB, IL-6, IL-1β, and TNF-α) and facilitates the infiltration of immune cells in animal models of ulcerative colitis [98,99,100]. In another study, it has been demonstrated that P2X7 receptor activation promotes intestinal inflammation in TNBS (2,4,6-trinitrobenzenesulfonic acid) and oxazolone-induced colitis, two models of chemically induced colitis. The study revealed that P2X7 receptor-deficient mice have a higher number of Tregs in the colonic lamina propria [101]. Furthermore, flow cytometry analysis of lymph nodes revealed that P2X7 receptor activation by ATP triggered death and retention of Tregs, thereby impairing gut immune tolerance [101]. This study provides substantial evidence in support of a significant role for the P2X7 receptor in the establishment of the enhanced inflammatory response during IBD, by promoting Treg cell death and compromising immune system tolerance in the gut [101].

Regarding P2Y receptors, increased expression of the P2Y_6_ receptor on epithelial cells has been associated with inflammation in DSS (dextran sodium sulfate)-induced mouse colitis [102]. However, another study highlighted a protective role for this receptor in regulating the quality of mucus in a DSS-induced colitis model. Indeed, it has been reported that P2Y_6_ receptor-deficient mice exhibited poor mucus quality and were more sensitive to DSS, resulting in increased disease activity index [103]. A study also demonstrated that deletion of P2Y_6_ receptor in mice exacerbated DSS-induced intestinal colitis by increasing the infiltration of Th17/Th1 cells and neutrophils in their colons, which correlated with increased levels of IFN-γ and IL-17A in the sera as well as increased mRNA levels of IFN-γ, IL-17A, IL-6, IL-23, and IL-1β in P2Y_6_ receptor-deficient colons [104]. However, it is not clear if P2Y_6_ receptor is expressed on Th1/Th17 cells and if its effect is direct or mediated through the dysregulation of immune tolerance mechanisms.

The importance of purinergic signaling in the regulation of IBD comes also from studies on ectonucleotidases. Indeed, the use of knockout mice showed in preclinical models that both CD39 and CD73 can protect from IBD likely through the generation of Tregs, thus limiting the expansion of pathogenic Th17 cells [105,106,107]. These pathways however seem to be dysregulated in patients and therefore therapeutic agents that can enhance the development of CD39^+^ Tregs could be considered as promising therapeutic strategy. This is the case with two agents, namely indole-3′-carbonyl-thiazole-4-carboxylic acid methyl ester (ITE) and unconjugated bilirubin [105,108,109]. Of note, treatment with GM-CSF, which was reported to reduce clinical symptoms of Crohn’ disease, led to the expression of CD39 and CD73 on activated monocytes which reduce inflammation in the context of DSS-induced colitis model, either directly by hydrolyzing pro-inflammatory extracellular ATP into adenosine or indirectly by promoting the formation of Tregs from naïve T cells [110].

The majority of studies on P2 receptors in IBD have been conducted in models of chemical-induced colitis in mice. It will be interesting to study these receptors in T cell-dependent models like IL-10 knock-out mice and the T cell transfer model.

### 5.5. Liver Autoimmunity

Autoimmune hepatitis (AIH) is an organ-specific autoimmune illness characterized by hypergammaglobulinemia, autoantibody positivity, and histological presence of interface hepatitis [111]. It is a severe liver disease caused by the abnormal activation of CD8^+^ and CD4^+^ effector T cells, including Th17 cells [112].

P2X4-deficient receptor mice have been utilized to investigate the role of this receptor in three distinct models of acute liver injury induced by concanavalin A (ConA), carbon tetrachloride, and acetaminophen [113]. The findings of the study revealed that ablation of the P2X4 receptor significantly reduced the severity of hepatitis in mice caused by ConA by restraining inflammation, oxidation, and cell death programs (apoptosis, autophagy, and NLRP3 inflammasome-activated pyroptosis [113]. Accordingly, ELISA analysis revealed increased levels of serum inflammatory mediators IL-1β, IL-6, IL-17A, IFN-γ, and TNF-α in ConA-treated WT mice when compared to P2X4 receptor-deficient mice [113]. Furthermore, treatment with the P2X4 receptor antagonist (5-BDBD) alleviated ConA-induced autoimmune hepatitis [113]. This study is the first to demonstrate that the absence of the P2X4 receptor may reduce immune-mediated liver damage, potentially by inhibiting inflammatory, oxidative, and programmed cell death processes [113]. The study also emphasizes that ConA-induced acute hepatitis requires the P2X4 receptor activation.

Research conducted with AIH patients has indicated functional impairment in CD39^+^ Treg cells. The authors demonstrated that Tregs expressing the ectonucleotidase CD39 are present at low levels and are also dysfunctional. Hence, these Tregs fail to hydrolyze pro-inflammatory ATP adequately, are unable to synthesize adenosine, and subsequently to inhibit Th17 cell immunity [114].

Table 1 below summarizes the established roles of P2 receptors and their function in autoimmune diseases.

## 6. Conclusions

This article provides a review of the role of P2 receptors in effector T cells and their implications in autoimmune disease. Thus, P2 receptors can promote effector T cells’ function either directly or by acting on the production of polarizing cytokines by antigen-presenting cells. On the other hand, P2 responses can also inhibit effector T cells and limit inflammatory response, mostly via dendritic cells or by enhancing Treg cells through CD39. Additional work is needed to further understand how P2 receptors affect effector T cells, especially with human models, and decipher how P2 receptors regulate the transcriptional programs of effector T cells. There are also some conflicting studies regarding the implication of P2 receptors in autoimmune diseases. This may be due to the use of different or less specific antagonists, or to variations in study models. Further studies are necessary to elucidate the role of purinergic signaling in effector T cells and the progression of autoimmune diseases by using more specific antagonists and uniformizing animal models.

Conversely, studies on the P2X4 receptor seem to be conclusive. Indeed, P2X4 has been implicated in autoimmune arthritis, lupus, and autoimmune hepatitis. This receptor also seems to be protective in the EAE model. Similarly, P2Y_6_ is protective in IBD and EAE. Clinical trials are necessary to determine whether these receptors constitute viable therapeutic targets in those autoimmune diseases.

Although evidence supports the involvement of purinergic signaling in autoimmune diseases, its relative importance compared to other intracellular pathways remains to be fully established despite known crosstalk with key signaling systems such as the TCR, chemokine receptors, and integrins, which play critical roles in autoimmunity. In addition, there is insufficient evidence to confirm whether purinergic signaling reliably correlates with disease activity in clinical settings. However, a decrease in extracellular ATP levels and an increase in its downstream metabolites, particularly adenosine and inosine, were frequently observed in multiple sclerosis, indicating a shift in metabolism toward an anti-inflammatory environment [115] suggesting the use of ATP and its metabolites as biomarkers for the disease. Finaly, and as discussed, data from preclinical models indicate that P2 receptors may exert either pro-inflammatory or protective effects, depending on the autoimmune disease context and the cell types involved. This suggests that purinergic signaling could have a context-dependent role, being protective in some diseases while promoting pathology in others.

Therefore, further mechanistic studies and well-designed clinical studies are necessary to clarify the precise role and diagnostic or prognostic value of purinergic signaling in autoimmune diseases.

Finally, it is important to emphasize that selective targeting of P2 receptors became a reality. Although the development of clinically viable drugs remains challenging due to pharmacokinetic limitations, receptor similarity, species differences, and the intricate nature of purinergic signaling, selective control of P2 receptors is now a realistic goal. P2Y_12_ antagonists, such as clopidogrel, are widely used as antiplatelet agents for the treatment of thrombosis and stroke [8]. Diquafosol, a P2Y_2_ receptor agonist, is approved for the treatment of dry eye disease in Japan and South Korea [8]. Additional drugs targeting other P2Y receptors such as P2Y_6_, P2Y_11_, P2Y_13_, and P2Y_14_ are currently in development [116].

Drug development targeting P2X receptors is more challenging due to the structural complexity of ion channels; however, a few promising candidates are in progress. These include a highly selective P2X7 antagonist being developed for the treatment of chronic kidney disease [117] and P2X3 antagonists such as Gefapixant, which has been FDA-approved for the treatment of refractory chronic cough, and Filapixant, which is undergoing clinical trials to assess its safety, tolerability, and pharmacokinetics [118].

Therefore, it is plausible that drugs targeting P2 receptors could also be used in the therapeutics of autoimmune diseases.

## Figures and Tables

**Table 1:** P2 receptors in autoimmune diseases.

P2 Receptor	Function	Autoimmune Disease	References
P2X4	Pathogenic: Increased IL-17 production by effector/memory CD4^+^ T cells Activation and differentiation of Th17 cells	Rheumatoid Arthritis	[34]
	Pathogenic: Promotion of Th17 activation and increased levels of IL-17 in arthritic joints	Collagen-induced Arthritis mouse model	[34]
	Pathogenic: Increased levels of serum of IL-1β, TNF-α, IL-6, and IL-17	Collagen-induced Arthritis mouse model	[78]
	Pathogenic: Increased Proteinuria (kidney disease) and autoantibody titers	NZB/W Mouse model of Systemic Lupus Erythematosus	[92]
	Pathogenic: Increased levels of serum inflammatory mediators IL-1β, IL-6, IL-17A, IFN-γ, and TNF-α.	Concanavalin-induced mouse model of Liver Autoimmunity	[113]
	Protective: Promotion of microglia remyelination	Myelin oligodendrocyte glycoprotein (MOG_35–55_)-induced mouse model of EAE	[94]
P2X7	Pathogenic: Upregulation of pro-inflammatory cytokines (Myd88, NF-κB, IL-6, IL-1β, and TNF-α) production	DSS-induced rat colitis	[98]
	Pathogenic: Promotion of Th17 differentiation and expression of Th17 polarizing cytokines (IL-1β, TGF-β1, IL-23p19, and IL-6)	Collagen-induced Arthritis mouse model	[22]
P2Y_6_	Protective: Reduction in Th1 and Th17 cells in the colon	DSS-induced mouse colitis	[104]
	Protective: Promotes mucus quality	DSS-induced mouse colitis	[103]
	Protective: Inhibition of the production of Th1 and Th17 polarizing cytokines (IL-12 and IL-23)	MOG_35–55_-induced mouse model of EAE	[53]

## Data Availability

No new data were created or analyzed in this study. Data sharing is not applicable to this article.
